# The effects of gender and age on health related behaviors

**DOI:** 10.1186/1471-2458-9-213

**Published:** 2009-06-30

**Authors:** Amanda Deeks, Catherine Lombard, Janet Michelmore, Helena Teede

**Affiliations:** 1 The Jean Hailes Foundation for Women's Health Research Unit, Monash Institute of Health Services Research, Kanooka Grove, Clayton, Victoria, Australia

## Abstract

**Background:**

Lifestyle-related diseases, including diabetes, cardiovascular disease, and some cancers represent the greatest global health threat. Greater insight into health needs and beliefs, using broad community samples, is vital to reduce the burden of chronic disease. This study aimed to investigate gender, age, screening practices, health beliefs, and perceived future health needs for healthy ageing.

**Methods:**

Random probability sampling using self-completion surveys in 1456 adults residing in Australia.

**Results:**

Screening behaviors were associated with gender and age. Men and women >51 years were more likely (27%) to have screening health checks than those <50 years (2%). Factors nominated to influence health were lifestyle (92%), relationships (82%), and environment (80%). Women were more likely to nominate preparedness to have an annual health check, willingness to seek advice from their medical practitioner and to attend education sessions. Numerous health fears were associated with ageing, however participants were more likely to have a financial (72%) rather than a health plan (42%). More women and participants >51 years wanted information regarding illness prevention than men or those aged <30 years.

**Conclusion:**

Age and gender are associated with health related behaviors. Optimal health is perceived as a priority, yet often this perception is not translated into preventative action. These findings will inform future research and policy makers as we strive towards a healthier ageing society and the prevention of chronic disease.

## Background

The greatest cause of mortality is lifestyle-related disease and illness, including diabetes (DM2), cardiovascular disease (CVD) and colon cancer [1-8]. Even though it is estimated that 90% of heart disease is preventable, in western populations we have failed to institute effective preventative lifestyle changes, have persisted with unhealthy lifestyles and are sedentary and increasingly overweight. To reduce the incidence and burden of chronic illness, it is vital to understand health related behaviors, including risk perception, particularly around preventative health care. This understanding should incorporate screening behaviors, health beliefs and perceived future needs for health.

Community chronic disease prevention practices, perceived responsibility for healthy lifestyle and disease prevention, health information needs, education opportunities and preparedness to undertake an active role in health have not been adequately explored. With regard to screening behaviors, past studies have evaluated specific practices; mammography or prostate checks, for example, or there has been a focus on physical activity or diet. Past research has also often been limited to one gender, or to specific age groups. There has been little population evaluation of health fears and behaviors associated with prevention of chronic disease in both men and women across the adult lifespan. Research in this area needs to take a broad population-wide approach to exploration of disease prevention, across a range of conditions, behaviors and lifestyle.

Previous literature on prevention has often been based on quality assurance data from the general practice setting. Steven et al 1999 found in a study of preventative care by general practitioners (GPs) that there is significant variability in reports of preventative health screening practices: BP checks (range from 36–100%), pap smears (range 10–91%) and lifestyle advice (9–10%) [9]. Screening can be opportunistic, driven by patients, and/or occur opportunistically with medical contact, or take a population-based approach (e.g. pap smears and mammograms). Lifestyle-related disease screening in most western countries is currently opportunistic and is heavily reliant on risk perception and on the beliefs of individuals. This highlights the need for greater research and understanding of behaviors and beliefs in these areas.

Risk perception motivates attendance to healthcare providers, promotes behavioral and lifestyle changes and influences decisions regarding treatments. An understanding of risk perception as it relates to prevention of chronic disease management is, therefore, important. Literature on risk perception and consequent behavior change (or not), is often limited to specific factors such as substance use including alcohol and smoking, sexual activity and transmitted disease, HIV, cancer risk, increasing physical activity and, more recently, the risk of SARS. Risk perception is complex and influenced by many factors, including the immediacy of the risk, the severity of consequences, knowledge of the risk, attitude, personal experience, personality, emotional wellbeing, the influence of affect and the social and cultural context. Due to the complexity of risk perception, before trying to understand why people may participate in health behaviors in order to reduce their risk of chronic disease, it will be helpful to understand what their priorities, beliefs and fears about future ill health and age might be.

Gender, age and sociocultural factors are likely to influence health related behavior including screening [10-12]. Women are more likely to attend for screening if they are 'health conscious', have had prior screening, or are aware, for example, that mammography reduces the risk of developing advanced breast cancer [13,14]. If women attend for mammography however, it is not known if they also attend for other screening tests (e.g. cholesterol). Often women become the family health managers, encouraging male partners and family members to attend for health checks. Exploration of gender differences in health behaviors is an important step towards an understanding of preventative practices and future education needs. Again, little is understood about age differences in screening practices and perceived needs as they relate to prevention of illness. It is also unclear whether individuals perceive that their health is their own responsibility, if they act on these perceptions, or if they rely on health professionals to implement health checks. What *is *known is that barriers to screening include the perceived need for a referral, lack of discussion with health care providers and unsatisfactory relationships with GPs [9,15-18].

The aim of the present study was to report on health beliefs, behaviors (including screening practices and preventative health practices), education and information needs, future health fears, and perceived responsibility relevant for improving health outcomes across gender and age groups. These data will contribute to the current evidence base to inform the development of educational programs at a population, community and individual level, targeting both general community and health professionals.

## Methods

The Jean Hailes Foundation for Women's Health is a national not for profit organisation focusing on the health and wellbeing of the five million Australian women aged 35 and over. Funded by the federal government, the organisation delivers evidence based information on women's health issues, including menopause. In total, 1126 self-completion surveys were sent to households throughout rural and urban Australia as part of the Pfizer Australia Health Report Series (see Additional file 1). Questionnaires were printed and distributed by post by an independent market research company, Stollznow Research, in April 2005. The study meets the Australian National Health and Medical Research Council (NHMRC) ethical guidelines on audits/surveys, which includes informed consent, confidentiality and de-identification of participation. Each household was assigned a unique identification number, and questionnaires were completed anonymously. Each participant received a $5 scratch lottery ticket for their participation. Results and preliminary analysis of the data were compiled independently by Stollznow Research and further data interpretation and manuscript preparation were completed independently by the authors.

The survey was entitled 'What does the future hold: Australians and ageing' and the specific aim was to investigate the opinions and habits of Australians relating to aspects of healthy ageing. This report focuses on data related to screening behaviors, health beliefs, responsibility for health, and perceived future health needs. Random probability sampling was used for this research project, based on 2001 census figures for population distribution between states and within states (i.e. metropolitan areas and rural/regional Australia). Recruitment of new panel households was done using telephone numbers randomly extracted from the White Pages on CD Rom (2004 edition). Questions were developed by The Jean Hailes Foundation for Women's Health research, medical and education staff, with consumer consultation. Closed questions were developed based on this expert consultation (the full questionnaire is available, see Additional file 1). Examples of the types of questions asked and associated relevant data are presented in Tables 1, 2, 3 and 4. Participants were asked whether they participated in regular health screening and if they visited their GP for an annual health check. Participants also answered questions relating to what they believed contributed to good health, the responsibility for health, future plans for wellbeing, and health conditions about which they would like more information.

**Table 1:** Participants who report regular screening health checks expressed as a percentage.

Screening health check	Male	Female	Total	<30 years	31–40 years	41–50 years	51–60 years	61–70 years	71+ years
Mammograms (regularly) women only		45	45	5	10	39	87***	88***	72***

Pap smears (regularly) women only		72	72	60	89***	77	80*	61	44

Prostate check (regularly) men only	43		43	9	9	45	68***	78***	92***

Cholesterol (regularly)	44	46	45	5	25	39	72***	74***	82***

Blood pressure (regularly)	60	72***	67	37	47	66	87***	87***	95***

Blood glucose (regularly)	38	43	41	8	16	38	65***	64***	81***

* P < 0.05

** P < 0.01

***P < 0.001

**Table 2:** Perceived factors that influence health priorities expressed as a percentage

Factors contributing to how long people live (expressed as %)	Male	Female	Total	<30 years	31–40 years	41–50 years	51–60 years	61–70 years	71+ years
Lifestyle (exercise & diet)	90	94**	92	87	97**	94	95	94	86

Genetics (history of longevity in the family)	85	90**	88	80	90	87	94**	88	89

Stable home life/relationships	80	84*	82	75	83	82	85	89*	83

Environmental factors	74	80*	77	75	81*	82*	81	70	64

Having disease prevention strategy including regular screening	64	73***	69	66	72	65	75*	69	68

Work environment/type of job	65	65	65	69	77***	67	68	55	39

Having satisfying hobbies	62	67	65	61	68	62	71*	70	58

None of these/don't know	4	2	3	7***	1	3	1	1	2

* P < 0.05

** P < 0.01

***P < 0.001

**Table 3:** Responsibility for disease prevention expressed as a percentage

Disease prevention strategies	Male	Female	Total	<30 years	31–40 years	41–50 years	51–60 years	61–70 years	71+ years
Already seek advice from GP on disease profile and prevention plan	13	12	12	4	3	8	21***	25***	30***

Already have annual health check	11	11	11	1	0	4	23***	29***	29***

Already attend education sessions on staying healthy and QOL	4	3	3	3	1	2	5	6	6

Already seek out and read health promotion material to stay healthy	8	11	9	3	3	11	15**	16**	16**

Already attend interactive sessions	4	3	4	2	0	4	8**	4	7*

Prepared to attend education sessions on staying healthy and QOL	41	54***	48	44	47	52	56*	47	42

Prepared to have annual health check (over 50 yrs)	79	83*	82	87*	94***	87*	72	66	69

Prepared to seek out and read health promotion material to help stay healthy	57	71***	65	67	66	66	66	60	60

Feel it is your responsibility to seek advice towards disease prevention	89	94***	92	88	91	94	97*	95	91

Prefer to attend interactive sessions over electronic/written information	41	50**	47	44	45	46	47	50	49

Prepared to seek advice from GP on disease profile and prevention plan	67	74**	71	75	78**	75	65	63	60

* P < 0.05

** P < 0.01

***P < 0.001

**Table 4:** Nominated plans in place for future needs in relation to health and wellbeing expressed as a percentage

What plans are in place for future health and wellbeing (expressed as %)	Male	Female	Total	<30 years	31–40 years	41–50 years	51–60 years	61–70 years	71+ years
A plan for financial security	76**	69	72	60	70	79**	80**	75	67

A plan for physical activity	31	31	31	19	20	29	45***	47***	55***

A plan for healthy living	41	43	42	28	31	39	58***	60***	66***

A plan for social activities	41	41	41	31	29	39	54***	57***	63***

* P < 0.05

** P < 0.01

***P < 0.001

### Statistical analysis

The sample was adjusted for age and residential location in order to weight regional areas according to each state and provide a representative sample of age groups as per the Australian census data: Thirteen percent (n = 191) were aged under 30 years, 18% (n = 257) were aged 31–40 years, 20% (n = 286) were 41–50 years, 22% (319) 51–60 years, 17% (253) 61–70 years, and 10% (150) were 71 years or older. Frequency data are reported along with chi-square tests showing p-values at and above the 95% confidence level. Chi-square tests were performed for both age and gender (against the total sample) using Survey Craft, an automated system of significance testing.

## Results

A total of 866 completed household surveys were obtained giving an overall household response rate of 77%. Fifty-six percent of respondents were women (n = 810) and 44% were men (n = 646). This paper reports on data related to both women's and men's responses.

### Screening practices

Women (72%) were more likely to have their BP checked compared to men (60%) (p < 0.001). Participants over 51 years of age were more likely than younger respondents to report participating in specific screening health checks including mammogram (p < 0.001), prostate check (p < 0.001), cholesterol (p < 0.001), BP (p < 0.001) and blood glucose (p < 0.001) (Table 1). The only health check younger women (31–40 years) participated in more than older women (61+ years), was regular pap smear screening (89%), whilst regular prostate checks were higher in men over 51 years (79%).

### Health beliefs

The most influential factor that was perceived to influence health was lifestyle (92%, n = 1223), followed by genetics (88%, n = 1162), home life and relationships (82%, n = 1086), environment (77%, n = 1018) and having a disease prevention strategy (69%, n = 914) (Table 2). Women were significantly more likely to nominate disease prevention strategy (p < 0.001), lifestyle (p < 0.01), genetics (p < 0.01), a stable home life and environment (p < 0.05) as influencing health than men. Younger participants were more likely to rate lifestyle (97%, p < 0.01) and work environment (77%, p < 0.05) as impacting on health and longevity, while older participants nominated a stable home life and relationships (89%) as important to longevity more than any other age group (p < 0.05).

### Health behaviors

A small number of participants advised that they undertook disease prevention strategies. These included seeking advice from their GP (12%, n = 165), attending education sessions (3%, n = 45), or reading health promotion material (9%, n = 124) (Table 3). Older participants were more likely to undergo an annual health check (27%, p < 0.001), and already sought out and read health promotion material (16%, p < 0.01) compared to younger participants. Women (94%) were more likely to feel it was their responsibility to seek advice on disease prevention compared to men (89%, p < 0.001), and were more likely to nominate that they would participate in health prevention strategies such as seeking out reading material (p < 0.001) (Table 3).

### Future beliefs, behaviors and needs

A number of fears were nominated in relation to ageing. These included fear of losing loved ones (88%, n = 1062), losing independence (77%, n = 949), physical (68%, n = 815) and mental health conditions (65%, n = 788), followed by financial concerns (64%, n = 769), living in a retirement home (45%, n = 554), loneliness (41%, n = 473) and appearance (34%, n = 369).

When asked what plans were already in action for future wellbeing, many participants reported that they already had a plan for financial security (72%, n = 886), however only 42% (n = 505) had established a plan for healthy living (Table 4). Participants over 51 years were significantly more likely to have plans in place for future health and wellbeing compared to those in younger age groups (p < 0.001).

When men and women were asked what conditions they would like more information on, there were significant differences between genders and age groups. Women wanted more information on diabetes (p < 0.01), obesity (p < 0.001), depression and anxiety (p < 0.001), whilst men only wanted more information on prostate cancer (p < 0.001). Significantly more men than women were not interested in receiving information on illness prevention (12% versus 6% respectively) (p < 0.001). Those under 30 years were significantly less interested in information on how to prevent illness (p < 0.01). Those over 51 years were significantly more likely to want information on heart disease (52%, p < 0.05), BP (45%, p < 0.05), bowel cancer (47%, p < 0.05), stroke (47%, p < 0.01) and high cholesterol (47%, p < 0.01).

## Discussion

The present study found that health behavior, and beliefs relating to responsibility for health and future health requirements, are associated with both gender and age. This is important data, informing on health related behavior which will contribute to educating and supporting the preventative fight against lifestyle related and chronic disease as we age.

In this study, differences between gender and age groups were reported across a range of health related behaviors including (1) screening behaviors (2) health beliefs and disease prevention strategies, influences and perceived responsibility and, (3) future needs for wellbeing. Those surveyed were aware of the need for change, reported readiness and acceptance of responsibility to change, yet did not translate this through optimal health related behavior. Important observations made in the current study are that participants believe their lifestyle and health are a top priority and they fear ill health more than financial problems, yet they plan more carefully for financial security than for continued health. It is clear that further understanding and research into preventative health, incorporating gender and age differences, is required.

It was disturbing to find that 40% of women under the age of 30 years did not present regularly for pap screening. In terms of population-based screening, data from the Australian Cervical Cytology Registry reflects that more than one third of women under the age of 30 do not present for pap smears, and since 1996 the percent of younger women being screened is decreasing [19]. American data from 1976 through to 2000 also confirm that the rate of cervical adenocarcinoma increased in young women during that period [20]. Herbert et al 2008 found delaying the age of cervical screening increases the risk of cervical cancer becoming more extensive [21]. Their recommendation was for the English health service to reverse the decision not to routinely screen women aged 20–24 years and that early pap tests provide an excellent opportunity for education on healthy lifestyles and safe sex practices. This finding is potentially related to the observation that younger participants are less likely to have annual health checks, seek advice or attend education sessions. Younger women in particular nominated that they were prepared to attend regular health checks, however the present study and one past study confirm they do not always act on this [20]. It would seem prudent for regular screening to begin at an earlier age. If preventative health care is to be effective it may be enhanced by opportunistic screening. Provision of support for medical professionals to accomplish this could be an important focus in formulating health policy. Perhaps a government-subsidised early adult health check (such as the Australian 45–49 year old health check) would also improve knowledge and screening behavior. This may in fact also be cost effective as a preventative measure against the potential expenses associated with chronic disease such as diabetes and CVD.

Obesity and adverse lifestyle are the primary risk factors for metabolic disorders and cardiovascular disease (CVD). The incidence of these conditions is increasing, yet they are preventable with sufficient lifestyle changes, as found with polycystic ovary syndrome for example [22]. Women are at an equal or greater risk of CVD than men, yet women incorrectly perceive they are more likely to die of breast cancer [23-25]. Education and communication of more accurate risk and of preventable risk factors, at both a community and individual level, is important. The knowledge that women in the present study would seek reading material, and would prefer to attend interactive education sessions is supportive of this as a preventative health measure. Further work is needed in this area.

Lifestyle was nominated as the most important factor in contributing to healthy ageing, followed by family history, a stable home life, environmental factors and having a disease prevention strategy in place. Different age groups nominated different factors as important across the life span. Education and preventative programs need to take account of these differences and priorities across life stages when targeting health related behaviors. For example, older participants nominated relationships as important to health and past research confirms that social support is important to emotional and physical wellbeing [26,27]. Armed with this knowledge, it may be prudent to develop age specific programs in the promotion of health; for example, offering social support programs for people in their sixties as a contribution to prevention of chronic disease.

Many participants in the present study reported that they would prefer to seek advice from their GP instead of from other sources. However, only 11% attended for preventative health screening. Previous research has shown that those adults who consult a GP on a regular basis were more likely to be satisfied with the preventative information and support given [9,15]. These and other factors need to be considered. Barriers to attendance need to be addressed, and initiatives and strategies to increase screening and prevention practices implemented.

Participants reported that their health was their own responsibility, a top priority in their lives, and that they feared ill health as they aged. However, few acted to improve their future health as they aged. Potentially, more needs to be done to move people on from the preparedness to take responsibility and the contemplation stage of change, into active participation in prevention strategies [28]. Learning and adapting from other successful ageing strategies (e.g. financial planning) could be applied to the health sector. Many governments mandate financial plans associated with retirement, and perhaps we must also start thinking about mandating certain health checks as an investment in future prevention. Providing free or low cost, regular interactive education seminars, may also promote more active participation in health behaviors.

Gender significantly influenced information seeking. Men were not as interested as women in diabetes, osteoporosis, eye conditions, obesity or mood disorders such as anxiety or depression. This is of particular concern given the growing number of diagnosed diabetes and CVD cases in the community. Women reported taking more responsibility for their health, potentially related to risk perception and the gender bias that women are socialized to be more concerned about health issues than men [10,12]. Targeted education, encouraging men to be engaged and to take more responsibility for their health will be vital to disease prevention.

There are some limitations with regard to the present study. This was a random sample representative of the Australian population, and as such may be expected to be representative of a broad cross-section of incomes and education levels. It would be beneficial for future research to more fully correct and evaluate the influence of socioeconomic factors on health behaviors in this population. Some participants had previously participated in health surveys in unrelated areas, which may or may not have influenced answers. There is also the possibility that response bias was present due to the method used to obtain data, including having a telephone and being available at the time of contact. The design of the present study was cross-sectional. It would also be useful to examine these health related beliefs and behaviors in a longitudinal study using standardized measures. The survey included closed questions and further information would be obtained by also providing participants in future studies with open-ended questions. As with all surveys, definitions and items can be open to interpretation.

## Conclusion

This study identifies both gender and age as factors which contribute to health related beliefs and behaviors. Although lifestyle and optimal health are believed to be a priority, many Australians do not translate this into preventative strategies such as regular screening. The western world needs to optimise recognition of health related behaviors, and, via public health initiatives, promote disease prevention and acceptance of health responsibility to individuals in order to tackle the growing burden of chronic disease.

## Competing interests

The authors declare that they have no competing interests.

*Financial support disclaimer: *As part of a series of surveys on health in Australia, "The Pfizer Australia Health Report", Pfizer Australia funded the costs generated from the independent marketing company, as part of their corporate social responsibility agenda. Pfizer Australia had no influence over the topic or questions asked and had no direct interest in the findings. The topic of research, development of questions and interpretation of data remain the intellectual property of The Jean Hailes Foundation for Women's Health. No authors have any financial interests or dealings with Pfizer Australia.

## Authors' contributions

AD contributed to study design and coordination, liaised with statistician and wrote the manuscript. CL provided intellectual input. JM contributed to study design and coordination. HT contributed to study design, provided intellectual input and contributed to manuscript. All authors read and approved the manuscript.

## Pre-publication history

The pre-publication history for this paper can be accessed here:



## Supplementary Material

Additional file 1 **The Jean Hailes Foundation for Women's Health: what does the future hold: Australians and ageing.** A health survey. A copy of the survey used. Click here for file
