# Supplementary material for: The effects of gender and age on health related behaviors
Source: BMC Public Health. 2009 Jun 30;9:213. doi: 10.1186/1471-2458-9-213 (PMC2713232; doi:10.1186/1471-2458-9-213)
Supplement: Additional file 1 — The Jean Hailes Foundation for Women's Health: what does the future hold: Australians and ageing. A health survey. A copy of the survey used. [file 1471-2458-9-213-S1.doc]

**The Jean Hailes Foundation for Women’s Health: What does the future hold: Australians and ageing**

| **HOW TO ANSWER THIS QUESTIONNAIRE** | | | | | | | | | | |
| --- | --- | --- | --- | --- | --- | --- | --- | --- | --- | --- |
|  | | | | | | | | | | |
|  | | | | | | | | | | |
| As discussed in the Introduction Sheet, we do not want you to use your last name on this questionnaire. We have identified you by your nominated name and ‘household number’. This means that no one who looks at this questionnaire can tell who answered it. Your nominated name is noted on the first page of the questionnaire together with a person number for your household, e.g. Person 1. | | | | | | | | | | |
|  | | | | | | | | | | |
| There are three types of questions in this questionnaire: | | | - Yes/No - Selected choice - Ranking. | | | | | | | |
|  | | | | | | | | | | |
| For each question, each household member is to **circle the number next to their response**—see sample below. | | | | | | | | | | |
|  | | | | | | | | | | |
| Please answer all questions according to the same given person number noted at the top of each page of the questionnaire. | | | | | | | | | | |
|  | | | | | | | | | | |
|  | | | | | | | | | | |
|  | |  | | *Person 6* |  |  |  |  |  |  |
|  | |  | | *Person 5* |  |  |  |  |  |  |
|  | |  | | *Person 4* |  |  |  |  |  |  |
|  | |  | | *Person 3* |  |  |  |  |  |  |
|  | |  | | *Person 2* |  |  |  |  |  |  |
|  | |  | | *Person 1* |  |  |  |  |  |  |
|  | | | | |  |  |  |  |  |  |
| **Q.1** | How would your rate your knowledge of glaucoma on this scale? | | | |  |  |  |  |  |  |
|  | Highly knowledgeable | | | | 1 | 1 | 1 | 1 | 1 | 1 |
|  | Some knowledge | | | | 2 | 2 | 2 | 2 | 2 | 2 |
|  | Little knowledge | | | | 3 | 3 | 3 | 3 | 3 | 3 |
|  | No knowledge at all | | | | 4 | 4 | 4 | 4 | 4 | 4 |
|  | | | | | | | | | | |
|  | | | | | | | | | | |
|  | | | | | | | | | | |
|  | | | | | | | | | | |

| **Jean Hailes Foundation**  **Healthy Aging and Emotional Wellbeing**. Australians are living longer than ever before, and the aim of the Jean Hailes Foundation is to empower and assist Australians to make these years healthy and to optimise our quality of life as we age.  **These questions are to be answered by all those in the household 16 years and over.** | | | | | | | | | | | | | | |  |  |  |  |  |  |
| --- | --- | --- | --- | --- | --- | --- | --- | --- | --- | --- | --- | --- | --- | --- | --- | --- | --- | --- | --- | --- |
| **Q.78** | Before reading this questionnaire, have you ever heard of the Jean Hailes Foundation? | | | | | | | | | | | | | |  |  |  |  |  |  |
|  |  | | | | | | | | | | | | | Yes | 1 | 1 | 1 | 1 | 1 | 1 |
|  |  | | | | | | | | | | | | | No | 2 | 2 | 2 | 2 | 2 | 2 |
| **Osteoporosis** | | | | | | | | | | | | | | |  |  |  |  |  |  |
| **Q.79** | How would you rate your knowledge of osteoporosis? | | | | | | Highly knowledgeable | | | | | | | | 1 | 1 | 1 | 1 | 1 | 1 |
|  |  |  |  |  |  |  | Some knowledge | | | | | | | | 2 | 2 | 2 | 2 | 2 | 2 |
|  |  | | | | | | Little knowledge | | | | | | | | 3 | 3 | 3 | 3 | 3 | 3 |
|  |  | | | | | | Not interested | | | | | | | | 4 | 4 | 4 | 4 | 4 | 4 |
|  |  | | Don’t know what osteoporosis is | | | | | | | | | | | | 5 | 5 | 5 | 5 | 5 | 5 |
|  | |  | | | | | | *Person 6* | | | | | | |  |  |  |  |  |  |
| For each question, each household member is to **circle the number next to their response** | |  | | | | | | *Person 5* | | | | | | |  |  |  |  |  |  |
|  |  |  | | | | | | *Person 4* | | | | | | |  |  |  |  |  |  |
|  |  |  | | | | | | *Person 3* | | | | | | |  |  |  |  |  |  |
| **Jean Hailes Foundation (continued)** | |  | | | | | | *Person 2* | | | | | | |  |  |  |  |  |  |
|  |  |  | | | | | | *Person 1* | | | | | | |  |  |  |  |  |  |
| **Osteoporosis (continued)** | | | | | | | | | | | | | | |  |  |  |  |  |  |
| **Q.80** | In which of the following ways can osteoporosis affect your health? (**SELECT ALL THAT APPLY**) | | | | | | | | | | | | | |  |  |  |  |  |  |
|  |  | | | | | | | | | | | | Pain | | 1 | 1 | 1 | 1 | 1 | 1 |
|  |  | | | Tiredness and general ill health | | | | | | | | | | | 2 | 2 | 2 | 2 | 2 | 2 |
|  |  | | | | | | | | | | | | Arthritis | | 3 | 3 | 3 | 3 | 3 | 3 |
|  |  | | | | | | | | | | | | Fractures | | 4 | 4 | 4 | 4 | 4 | 4 |
|  |  | | | | | | | | | | Poor mobility | | | | 5 | 5 | 5 | 5 | 5 | 5 |
|  |  | | | | Increased heart disease | | | | | | | | | | 6 | 6 | 6 | 6 | 6 | 6 |
|  |  | | | | | | | | | None of these | | | | | 7 | 7 | 7 | 7 | 7 | 7 |
|  |  | | | | | | | | | Don’t know | | | | | 8 | 8 | 8 | 8 | 8 | 8 |
| **Q.81** | Which of these can contribute to osteoporosis? (**SELECT ALL THAT APPLY**) | | | | | | | | | | | | | |  |  |  |  |  |  |
|  |  | | | | | | | | | | | | Age | | 1 | 1 | 1 | 1 | 1 | 1 |
|  |  | | | | | | | | Low dietary calcium | | | | | | 2 | 2 | 2 | 2 | 2 | 2 |
|  |  | | | | | Inadequate sun exposure | | | | | | | | | 3 | 3 | 3 | 3 | 3 | 3 |
|  |  | | | | | | | | Inadequate exercise | | | | | | 4 | 4 | 4 | 4 | 4 | 4 |
|  |  | | | | | | | | | | | | Smoking | | 5 | 5 | 5 | 5 | 5 | 5 |
|  |  | | | | | | | | | | | | Pain | | 6 | 6 | 6 | 6 | 6 | 6 |
|  |  | | | | | | | | | None of these | | | | | 7 | 7 | 7 | 7 | 7 | 7 |
|  |  | | | | | | | | | Don’t know | | | | | 8 | 8 | 8 | 8 | 8 | 8 |
| **Q.82** | At what age do you think people usually get symptoms related to osteoporosis? | | | | | | | | | | | | | |  |  |  |  |  |  |
|  |  | | | | | | | | In their 70s and 80s | | | | | | 1 | 1 | 1 | 1 | 1 | 1 |
|  |  | | | | | | | | | | | In their 60s | | | 2 | 2 | 2 | 2 | 2 | 2 |
|  |  | | | | | | | | | | | In their 50s | | | 3 | 3 | 3 | 3 | 3 | 3 |
|  |  | | | | | | | | | | | In their 40s | | | 4 | 4 | 4 | 4 | 4 | 4 |
|  |  | | | | | | | | | | | In their 30s | | | 5 | 5 | 5 | 5 | 5 | 5 |
|  |  | | | | | | | | | | | Under 30 | | | 6 | 6 | 6 | 6 | 6 | 6 |
|  |  | | | | | | | | | | | Any age | | | 7 | 7 | 7 | 7 | 7 | 7 |
|  |  | | | | | | | | | | | Don’t know | | | 8 | 8 | 8 | 8 | 8 | 8 |

|  | | |  | | | *Person 6* | | | |  |  |  |  |  |  |
| --- | --- | --- | --- | --- | --- | --- | --- | --- | --- | --- | --- | --- | --- | --- | --- |
| For each question, each household member is to **circle the number next to their response** | | |  | | | *Person 5* | | | |  |  |  |  |  |  |
|  |  |  |  | | | *Person 4* | | | |  |  |  |  |  |  |
|  |  |  |  | | | *Person 3* | | | |  |  |  |  |  |  |
| **Jean Hailes Foundation (continued)** | | |  | | | *Person 2* | | | |  |  |  |  |  |  |
|  |  |  |  | | | *Person 1* | | | |  |  |  |  |  |  |
| **Osteoporosis (continued)** | | | | | | | | | |  |  |  |  |  |  |
| **Q.83** | If you wanted to learn more about osteoporosis, which information source would you use? (**SELECT ALL THAT APPLY**) | | | | | | | | |  |  |  |  |  |  |
|  |  | | | | | | | GP | | 1 | 1 | 1 | 1 | 1 | 1 |
|  |  | | | | | | | Pharmacist | | 2 | 2 | 2 | 2 | 2 | 2 |
|  |  | | | | | | | Internet | | 3 | 3 | 3 | 3 | 3 | 3 |
|  |  | | | | Jean Hailes website | | | | | 4 | 4 | 4 | 4 | 4 | 4 |
|  |  | | | | Osteoporosis Australia | | | | | 5 | 5 | 5 | 5 | 5 | 5 |
|  |  | | | | | | | Magazines | | 6 | 6 | 6 | 6 | 6 | 6 |
|  |  | | | | | | | Newspapers | | 7 | 7 | 7 | 7 | 7 | 7 |
|  |  | | | Friend or family member | | | | | | 8 | 8 | 8 | 8 | 8 | 8 |
|  |  | Attend an education session in my local area if available | | | | | | | | 9 | 9 | 9 | 9 | 9 | 9 |
|  |  | | | | | | None of these | | | 10 | 10 | 10 | 10 | 10 | 10 |
| **Screening** | | | | | | | | | |  |  |  |  |  |  |
| **Q.84** | Do you have the following done regularly? | | | | | | | |  |  |  |  |  |  |  |
|  | Mammograms (at least two yearly in women)? | | | | | | | | Yes | 1 | 1 | 1 | 1 | 1 | 1 |
|  |  |  |  |  |  |  |  |  | No | 2 | 2 | 2 | 2 | 2 | 2 |
|  |  | | | | | | | |  |  |  |  |  |  |  |
|  | Pap smears (at least two yearly in women)? | | | | | | | | Yes | 1 | 1 | 1 | 1 | 1 | 1 |
|  |  |  |  |  |  |  |  |  | No | 2 | 2 | 2 | 2 | 2 | 2 |
|  |  | | | | | | | |  |  |  |  |  |  |  |
|  | Cholesterol check (at least two yearly over 50 years)? | | | | | | | | Yes | 1 | 1 | 1 | 1 | 1 | 1 |
|  |  |  |  |  |  |  |  |  | No | 2 | 2 | 2 | 2 | 2 | 2 |
|  |  | | | | | | | |  |  |  |  |  |  |  |
|  | Blood pressure check (at least 12 monthly over 50 years)? | | | | | | | | Yes | 1 | 1 | 1 | 1 | 1 | 1 |
|  |  |  |  |  |  |  |  |  | No | 2 | 2 | 2 | 2 | 2 | 2 |
|  |  | | | | | | | |  |  |  |  |  |  |  |
|  | Blood sugar levels (at least two yearly over 55 years) | | | | | | | | Yes | 1 | 1 | 1 | 1 | 1 | 1 |
|  |  |  |  |  |  |  |  |  | No | 2 | 2 | 2 | 2 | 2 | 2 |
|  |  | | | | | | | |  |  |  |  |  |  |  |
|  | Prostate checks (men only—have you ever had a check)? | | | | | | | | Yes | 1 | 1 | 1 | 1 | 1 | 1 |
|  |  |  |  |  |  |  |  |  | No | 2 | 2 | 2 | 2 | 2 | 2 |
|  |  | | | | | | | |  |  |  |  |  |  |  |
|  | Glaucoma check (at least one by age 40) | | | | | | | | Yes | 1 | 1 | 1 | 1 | 1 | 1 |
|  |  |  |  |  |  |  |  |  | No | 2 | 2 | 2 | 2 | 2 | 2 |

|  | |  | *Person 6* | | |  |  |  |  |  |  |
| --- | --- | --- | --- | --- | --- | --- | --- | --- | --- | --- | --- |
| For each question, each household member is to **circle the number next to their response** | |  | *Person 5* | | |  |  |  |  |  |  |
|  |  |  | *Person 4* | | |  |  |  |  |  |  |
|  |  |  | *Person 3* | | |  |  |  |  |  |  |
| **Jean Hailes Foundation (continued)** | |  | *Person 2* | | |  |  |  |  |  |  |
|  |  |  | *Person 1* | | |  |  |  |  |  |  |
| **Screening (continued)** | | | | | |  |  |  |  |  |  |
| **Q.85** | Would you be prepared to do the following? | | | |  |  |  |  |  |  |  |
|  | Have an annual health check every one to two years over the age of 50 years? | | | |  |  |  |  |  |  |  |
|  |  | | | | Yes | 1 | 1 | 1 | 1 | 1 | 1 |
|  |  | | | | No | 2 | 2 | 2 | 2 | 2 | 2 |
|  |  | | | Already do this | | 3 | 3 | 3 | 3 | 3 | 3 |
|  | Seek advice from your GP on your disease risk profile and illness prevention plan? | | | |  |  |  |  |  |  |  |
|  |  | | | | Yes | 1 | 1 | 1 | 1 | 1 | 1 |
|  |  | | | | No | 2 | 2 | 2 | 2 | 2 | 2 |
|  |  | | | Already do this | | 3 | 3 | 3 | 3 | 3 | 3 |
|  | Attend a series of education sessions in your local area on how to stay healthy and improve your quality of life as you age? | | | |  |  |  |  |  |  |  |
|  |  | | | | Yes | 1 | 1 | 1 | 1 | 1 | 1 |
|  |  | | | | No | 2 | 2 | 2 | 2 | 2 | 2 |
|  |  | | | Already do this | | 3 | 3 | 3 | 3 | 3 | 3 |
|  | Seek out and read health promotion material to help you stay healthy? | | | |  |  |  |  |  |  |  |
|  |  | | | | Yes | 1 | 1 | 1 | 1 | 1 | 1 |
|  |  | | | | No | 2 | 2 | 2 | 2 | 2 | 2 |
|  |  | | | Already do this | | 3 | 3 | 3 | 3 | 3 | 3 |
|  | [**IF YES**] Would you prefer interactive sessions to electronic or written information? | | | |  |  |  |  |  |  |  |
|  |  | | | | Yes | 1 | 1 | 1 | 1 | 1 | 1 |
|  |  | | | | No | 2 | 2 | 2 | 2 | 2 | 2 |
|  |  | | | Already do this | | 3 | 3 | 3 | 3 | 3 | 3 |

|  | | | |  | | | *Person 6* | | |  |  |  |  |  |  |
| --- | --- | --- | --- | --- | --- | --- | --- | --- | --- | --- | --- | --- | --- | --- | --- |
| For each question, each household member is to **circle the number next to their response** | | | |  | | | *Person 5* | | |  |  |  |  |  |  |
|  |  |  |  |  | | | *Person 4* | | |  |  |  |  |  |  |
|  |  |  |  |  | | | *Person 3* | | |  |  |  |  |  |  |
| **Jean Hailes Foundation (continued)** | | | |  | | | *Person 2* | | |  |  |  |  |  |  |
|  |  |  |  |  | | | *Person 1* | | |  |  |  |  |  |  |
| **Screening (continued)** | | | | | | | | | |  |  |  |  |  |  |
| **Q.86** | | Would you be interested in further information on any of the following conditions on how to look after your health and stay healthy? | | | | | | | |  |  |  |  |  |  |
|  | |  | | | | | | Heart disease? | | 1 | 1 | 1 | 1 | 1 | 1 |
|  | |  | | | | | | Stroke? | | 2 | 2 | 2 | 2 | 2 | 2 |
|  | |  | | | | | | Diabetes? | | 3 | 3 | 3 | 3 | 3 | 3 |
|  | |  | | | | | | Osteoporosis? | | 4 | 4 | 4 | 4 | 4 | 4 |
|  | |  | | | | | | Prostate cancer? | | 5 | 5 | 5 | 5 | 5 | 5 |
|  | |  | | | | | | Breast cancer? | | 6 | 6 | 6 | 6 | 6 | 6 |
|  | |  | | | | | | Bowel cancer? | | 7 | 7 | 7 | 7 | 7 | 7 |
|  | |  | | | | | | Cervical cancer? | | 8 | 8 | 8 | 8 | 8 | 8 |
|  | |  | | | | | | Obesity? | | 9 | 9 | 9 | 9 | 9 | 9 |
|  | |  | | | | | | Blood pressure? | | 10 | 10 | 10 | 10 | 10 | 10 |
|  | |  | | | | | | High cholesterol? | | 11 | 11 | 11 | 11 | 11 | 11 |
|  | |  | | | | | | Depression? | | 12 | 12 | 12 | 12 | 12 | 12 |
|  | |  | | | | | | Anxiety? | | 13 | 13 | 13 | 13 | 13 | 13 |
|  | |  | | | | Eye conditions such as glaucoma? | | | | 14 | 14 | 14 | 14 | 14 | 14 |
|  | |  | | | | | | None of these? | | 15 | 15 | 15 | 15 | 15 | 15 |
|  | |  | Not interested in information on how to prevent illness | | | | | | | 16 | 16 | 16 | 16 | 16 | 16 |
| **Q.87** | | Do you feel it is your responsibility to seek advice on, and to work towards, disease prevention? | | | | | | |  |  |  |  |  |  |  |
|  | |  | | | | | | | Yes | 1 | 1 | 1 | 1 | 1 | 1 |
|  | |  | | | | | | | No | 2 | 2 | 2 | 2 | 2 | 2 |
| **Lifestyle** | | | | | | | | | |  |  |  |  |  |  |
| **Q.88** | Do you consider yourself a healthy weight? | | | | | | | |  |  |  |  |  |  |  |
|  |  | | | | | | | | Yes | 1 | 1 | 1 | 1 | 1 | 1 |
|  |  | | | | No, I consider that I am underweight | | | | | 2 | 2 | 2 | 2 | 2 | 2 |
|  |  | | | | No, I consider that I am overweight | | | | | 3 | 3 | 3 | 3 | 3 | 3 |

|  | | | |  | | *Person 6* | | |  |  |  |  |  |  |
| --- | --- | --- | --- | --- | --- | --- | --- | --- | --- | --- | --- | --- | --- | --- |
| For each question, each household member is to **circle the number next to their response** | | | |  | | *Person 5* | | |  |  |  |  |  |  |
|  |  |  |  |  | | *Person 4* | | |  |  |  |  |  |  |
|  |  |  |  |  | | *Person 3* | | |  |  |  |  |  |  |
| **Jean Hailes Foundation (continued)** | | | |  | | *Person 2* | | |  |  |  |  |  |  |
|  |  |  |  |  | | *Person 1* | | |  |  |  |  |  |  |
| **Lifestyle (continued)** | | | | | | | | |  |  |  |  |  |  |
| **Q.89** | Regarding your body weight, if you wanted to learn more about diet options, which information source would you use? | | | | | | |  |  |  |  |  |  |  |
|  | (**SELECT ALL THAT APPLY**) | | | | | | GP | | 1 | 1 | 1 | 1 | 1 | 1 |
|  |  | | | | | | Pharmacist | | 2 | 2 | 2 | 2 | 2 | 2 |
|  |  | | | | | | Internet | | 3 | 3 | 3 | 3 | 3 | 3 |
|  |  | Attend an education session in my local area if available | | | | | | | 4 | 4 | 4 | 4 | 4 | 4 |
|  |  | | | | | | Magazines | | 5 | 5 | 5 | 5 | 5 | 5 |
|  |  | | | | | | Newspapers | | 6 | 6 | 6 | 6 | 6 | 6 |
|  |  | | Weight loss organisations (Weight Watchers etc.) | | | | | | 7 | 7 | 7 | 7 | 7 | 7 |
|  |  | | | | | | Dietitians | | 8 | 8 | 8 | 8 | 8 | 8 |
|  |  | | | | Friends or family members | | | | 9 | 9 | 9 | 9 | 9 | 9 |
|  |  | | | | | | None of these | | 10 | 10 | 10 | 10 | 10 | 10 |
|  |  | | | | | | Don’t know | | 11 | 11 | 11 | 11 | 11 | 11 |
| **Q.90** | What are your levels of physical activity? | | | | | | |  |  |  |  |  |  |  |
|  | Walking—more than 20 minutes on a daily basis | | | | | | | Yes | 1 | 1 | 1 | 1 | 1 | 1 |
|  |  |  |  |  |  |  |  | No | 2 | 2 | 2 | 2 | 2 | 2 |
|  |  | | | | | | |  |  |  |  |  |  |  |
|  | Weekly gardening—more than 30 minutes weekly | | | | | | | Yes | 1 | 1 | 1 | 1 | 1 | 1 |
|  |  |  |  |  |  |  |  | No | 2 | 2 | 2 | 2 | 2 | 2 |
|  |  | | | | | | |  |  |  |  |  |  |  |
|  | Doing active housework, i.e vacuuming—more than 30 minutes weekly | | | | | | | Yes | 1 | 1 | 1 | 1 | 1 | 1 |
|  |  |  |  |  |  |  |  | No | 2 | 2 | 2 | 2 | 2 | 2 |
|  |  | | | | | | |  |  |  |  |  |  |  |
|  | Additional sporting activities—once per week | | | | | | | Yes | 1 | 1 | 1 | 1 | 1 | 1 |
|  |  |  |  |  |  |  |  | No | 2 | 2 | 2 | 2 | 2 | 2 |
|  |  | | | | | | |  |  |  |  |  |  |  |
|  | Additional sporting activities—more than once per week | | | | | | | Yes | 1 | 1 | 1 | 1 | 1 | 1 |
|  |  |  |  |  |  |  |  | No | 2 | 2 | 2 | 2 | 2 | 2 |
| **Q.91** | Do you consume on average— | | | | | | |  |  |  |  |  |  |  |
|  | More than three cups of coffee or tea daily? | | | | | | | Yes | 1 | 1 | 1 | 1 | 1 | 1 |
|  |  |  |  |  |  |  |  | No | 2 | 2 | 2 | 2 | 2 | 2 |
|  |  | | | | | | |  |  |  |  |  |  |  |
|  | One or more soft drinks daily? | | | | | | | Yes | 1 | 1 | 1 | 1 | 1 | 1 |
|  |  |  |  |  |  |  |  | No | 2 | 2 | 2 | 2 | 2 | 2 |

|  | |  | *Person 6* | | |  |  |  |  |  |  |
| --- | --- | --- | --- | --- | --- | --- | --- | --- | --- | --- | --- |
| For each question, each household member is to **circle the number next to their response** | |  | *Person 5* | | |  |  |  |  |  |  |
|  |  |  | *Person 4* | | |  |  |  |  |  |  |
|  |  |  | *Person 3* | | |  |  |  |  |  |  |
| **Jean Hailes Foundation (continued)** | |  | *Person 2* | | |  |  |  |  |  |  |
|  |  |  | *Person 1* | | |  |  |  |  |  |  |
| **Lifestyle (continued)** | | | | | |  |  |  |  |  |  |
| **Q.91** | Do you consume on average— | | | |  |  |  |  |  |  |  |
| **(ctd)** | More than one alcoholic drink daily? | | | | Yes | 1 | 1 | 1 | 1 | 1 | 1 |
|  |  |  |  |  | No | 2 | 2 | 2 | 2 | 2 | 2 |
|  |  | | | |  |  |  |  |  |  |  |
|  | Two to three serves of dairy food daily? (A serve is a glass of milk, a piece of cheese, a tub of yoghurt) | | | | Yes | 1 | 1 | 1 | 1 | 1 | 1 |
|  |  |  |  |  | No | 2 | 2 | 2 | 2 | 2 | 2 |
|  |  | | | |  |  |  |  |  |  |  |
|  | Fish two to three times per week? | | | | Yes | 1 | 1 | 1 | 1 | 1 | 1 |
|  |  |  |  |  | No | 2 | 2 | 2 | 2 | 2 | 2 |
|  |  | | | |  |  |  |  |  |  |  |
|  | Lean red meat three to four times per week? | | | | Yes | 1 | 1 | 1 | 1 | 1 | 1 |
|  |  |  |  |  | No | 2 | 2 | 2 | 2 | 2 | 2 |
|  |  | | | |  |  |  |  |  |  |  |
|  | Four serves of vegetables most days? | | | | Yes | 1 | 1 | 1 | 1 | 1 | 1 |
|  |  |  |  |  | No | 2 | 2 | 2 | 2 | 2 | 2 |
|  |  | | | |  |  |  |  |  |  |  |
|  | Two pieces of fruit most days? | | | | Yes | 1 | 1 | 1 | 1 | 1 | 1 |
|  |  |  |  |  | No | 2 | 2 | 2 | 2 | 2 | 2 |
| **Emotional wellbeing and relationships**  **If you have a partner/spouse please answer questions 92–94** | | | | | |  |  |  |  |  |  |
| **Q.92** | Are you satisfied with your relationship with your partner/spouse? | | | | |  |  |  |  |  |  |
|  |  | | | Very satisfied | | 1 | 1 | 1 | 1 | 1 | 1 |
|  |  | | | Mostly satisfied | | 2 | 2 | 2 | 2 | 2 | 2 |
|  |  | | | Somewhat satisfied | | 3 | 3 | 3 | 3 | 3 | 3 |
|  |  | | | Not really satisfied | | 4 | 4 | 4 | 4 | 4 | 4 |
| **Q.93** | How long have you been in your current relationship? | | | |  |  |  |  |  |  |  |
|  |  | | | Less than one year | | 1 | 1 | 1 | 1 | 1 | 1 |
|  |  | | | One to five years | | 2 | 2 | 2 | 2 | 2 | 2 |
|  |  | | | Six to 15 years | | 3 | 3 | 3 | 3 | 3 | 3 |
|  |  | | | 16 to 24 years | | 4 | 4 | 4 | 4 | 4 | 4 |
|  |  | | | Over 25 years | | 5 | 5 | 5 | 5 | 5 | 5 |

|  | |  | | *Person 6* | | | | | |  |  |  |  |  |  |
| --- | --- | --- | --- | --- | --- | --- | --- | --- | --- | --- | --- | --- | --- | --- | --- |
| For each question, each household member is to **circle the number next to their response** | |  | | *Person 5* | | | | | |  |  |  |  |  |  |
|  |  |  | | *Person 4* | | | | | |  |  |  |  |  |  |
|  |  |  | | *Person 3* | | | | | |  |  |  |  |  |  |
| **Jean Hailes Foundation (continued)** | |  | | *Person 2* | | | | | |  |  |  |  |  |  |
|  |  |  | | *Person 1* | | | | | |  |  |  |  |  |  |
| **Emotional wellbeing and relationships (continued)**  **If you have a partner/spouse please answer questions 94–96 also** | | | | | | | | | |  |  |  |  |  |  |
| **Q.94** | Do you— | | | | | | | |  |  |  |  |  |  |  |
|  | Find yourself just too busy to spend time nurturing your relationship? | | | | | | | | Yes | 1 | 1 | 1 | 1 | 1 | 1 |
|  |  |  |  |  |  |  |  |  | No | 2 | 2 | 2 | 2 | 2 | 2 |
|  |  | | | | | | | |  |  |  |  |  |  |  |
|  | Spend intimate time alone with your partner on a regular basis? | | | | | | | | Yes | 1 | 1 | 1 | 1 | 1 | 1 |
|  |  |  |  |  |  |  |  |  | No | 2 | 2 | 2 | 2 | 2 | 2 |
|  |  | | | | | | | |  |  |  |  |  |  |  |
|  | Make a specific effort to maintain intimacy? | | | | | | | | Yes | 1 | 1 | 1 | 1 | 1 | 1 |
|  |  |  |  |  |  |  |  |  | No | 2 | 2 | 2 | 2 | 2 | 2 |
|  |  | | | | | | | |  |  |  |  |  |  |  |
|  | Feel work, family and everyday life interferes with your time alone with your partner? | | | | | | | | Yes | 1 | 1 | 1 | 1 | 1 | 1 |
|  |  |  |  |  |  |  |  |  | No | 2 | 2 | 2 | 2 | 2 | 2 |
|  |  | | | | | | | |  |  |  |  |  |  |  |
| **Q.95** | Do you feel your sex life with your partner is: | | | | | | Satisfying | | | 1 | 1 | 1 | 1 | 1 | 1 |
|  |  | | | | | | Unsatisfying | | | 2 | 2 | 2 | 2 | 2 | 2 |
|  |  | | | | | | Non-existent | | | 3 | 3 | 3 | 3 | 3 | 3 |
|  |  | | Do not wish to answer this question | | | | | | | 4 | 4 | 4 | 4 | 4 | 4 |
| **Q.96** | Do you talk to your partner about intimate relationship issues or sexuality issues? | | | | | | | | |  |  |  |  |  |  |
|  |  | | | | | | | Freely | | 1 | 1 | 1 | 1 | 1 | 1 |
|  |  | | | | With some difficulty | | | | | 2 | 2 | 2 | 2 | 2 | 2 |
|  |  | | | | With great difficulty | | | | | 3 | 3 | 3 | 3 | 3 | 3 |
|  |  | | | | | | | Never | | 4 | 4 | 4 | 4 | 4 | 4 |
|  |  | | Do not wish to answer this question | | | | | | | 5 | 5 | 5 | 5 | 5 | 5 |
| **Q.97** | Which of these best describes your work status? | | | | | | | | |  |  |  |  |  |  |
|  |  | | | | | Student | | | | 1 | 1 | 1 | 1 | 1 | 1 |
|  |  | | | | | Unemployed | | | | 2 | 2 | 2 | 2 | 2 | 2 |
|  |  | | | | | Working part-time | | | | 3 | 3 | 3 | 3 | 3 | 3 |
|  |  | | | | | Working full-time | | | | 4 | 4 | 4 | 4 | 4 | 4 |
|  |  | | | | | Retired | | | | 5 | 5 | 5 | 5 | 5 | 5 |

|  | |  | | | | | *Person 6* | | | |  |  |  |  |  |  |
| --- | --- | --- | --- | --- | --- | --- | --- | --- | --- | --- | --- | --- | --- | --- | --- | --- |
| For each question, each household member is to **circle the number next to their response** | |  | | | | | *Person 5* | | | |  |  |  |  |  |  |
|  |  |  | | | | | *Person 4* | | | |  |  |  |  |  |  |
|  |  |  | | | | | *Person 3* | | | |  |  |  |  |  |  |
| **Jean Hailes Foundation (continued)** | |  | | | | | *Person 2* | | | |  |  |  |  |  |  |
|  |  |  | | | | | *Person 1* | | | |  |  |  |  |  |  |
| **Emotional wellbeing and relationships (continued)** | | | | | | | | | | |  |  |  |  |  |  |
| **Q.98** | What education level have you attained? | | | | | | | | | |  |  |  |  |  |  |
|  |  | | | | | Postgraduate studies | | | | | 1 | 1 | 1 | 1 | 1 | 1 |
|  |  | | | | University degree or equivalent | | | | | | 2 | 2 | 2 | 2 | 2 | 2 |
|  |  | | | Trade or business certificate/TAFE | | | | | | | 3 | 3 | 3 | 3 | 3 | 3 |
|  |  | High School Certificate (Year 12) or equivalent | | | | | | | | | 4 | 4 | 4 | 4 | 4 | 4 |
|  |  | | School Certificate (Year 10) or equivalent | | | | | | | | 5 | 5 | 5 | 5 | 5 | 5 |
|  |  | | | | | | | | | Other | 6 | 6 | 6 | 6 | 6 | 6 |
| **Q.99** | How many adults do you think experience anxiety or depression in Australia? | | | | | | | | | |  |  |  |  |  |  |
|  |  | | | | | | | One in two people | | | 1 | 1 | 1 | 1 | 1 | 1 |
|  |  | | | | | | | One in five people | | | 2 | 2 | 2 | 2 | 2 | 2 |
|  |  | | | | | | | One in ten people | | | 3 | 3 | 3 | 3 | 3 | 3 |
|  |  | | | | | | | One in 20 people | | | 4 | 4 | 4 | 4 | 4 | 4 |
| **Q.100** | Regarding depression or anxiety? | | | | | | | | | |  |  |  |  |  |  |
|  | Has your GP ever told you that you were depressed? | | | | | | | | | Yes | 1 | 1 | 1 | 1 | 1 | 1 |
|  |  |  |  |  |  |  |  |  |  | No | 2 | 2 | 2 | 2 | 2 | 2 |
|  |  | | | | | | | | |  |  |  |  |  |  |  |
|  | Have you ever been on medications specifically for depression? | | | | | | | | | Yes | 1 | 1 | 1 | 1 | 1 | 1 |
|  |  |  |  |  |  |  |  |  |  | No | 2 | 2 | 2 | 2 | 2 | 2 |
|  |  | | | | | | | | |  |  |  |  |  |  |  |
|  | Has your GP ever told you that you have an anxiety problem? | | | | | | | | | Yes | 1 | 1 | 1 | 1 | 1 | 1 |
|  |  |  |  |  |  |  |  |  |  | No | 2 | 2 | 2 | 2 | 2 | 2 |
|  |  | | | | | | | | |  |  |  |  |  |  |  |
|  | Are you concerned about anxiety and depression in future? | | | | | | | | | Yes | 1 | 1 | 1 | 1 | 1 | 1 |
|  |  |  |  |  |  |  |  |  |  | No | 2 | 2 | 2 | 2 | 2 | 2 |
|  |  | | | | | | | | |  |  |  |  |  |  |  |
|  | Would you be interested in finding out more about depression and anxiety problems? | | | | | | | | | Yes | 1 | 1 | 1 | 1 | 1 | 1 |
|  |  |  |  |  |  |  |  |  |  | No | 2 | 2 | 2 | 2 | 2 | 2 |
| **Q.101** | When do you plan to retire? | | | | | | | | | |  |  |  |  |  |  |
|  |  | | | | | | | | Aged 55 | | 1 | 1 | 1 | 1 | 1 | 1 |
|  |  | | | | | | | | 56–60 years | | 2 | 2 | 2 | 2 | 2 | 2 |
|  |  | | | | | | | | 61–65 years | | 3 | 3 | 3 | 3 | 3 | 3 |
|  |  | | | | | | | | Over 65 years | | 4 | 4 | 4 | 4 | 4 | 4 |

|  | |  | | *Person 6* | | |  |  |  |  |  |  |
| --- | --- | --- | --- | --- | --- | --- | --- | --- | --- | --- | --- | --- |
| For each question, each household member is to **circle the number next to their response** | |  | | *Person 5* | | |  |  |  |  |  |  |
|  |  |  | | *Person 4* | | |  |  |  |  |  |  |
|  |  |  | | *Person 3* | | |  |  |  |  |  |  |
| **Jean Hailes Foundation (continued)** | |  | | *Person 2* | | |  |  |  |  |  |  |
|  |  |  | | *Person 1* | | |  |  |  |  |  |  |
| **Emotional wellbeing and relationships (continued)** | | | | | | |  |  |  |  |  |  |
| **Q.102** | Do you have a retirement plan for: | | | | | |  |  |  |  |  |  |
|  | Financial security—superannuation | | | | | Yes | 1 | 1 | 1 | 1 | 1 | 1 |
|  |  |  |  |  |  | No | 2 | 2 | 2 | 2 | 2 | 2 |
|  |  | | | | |  |  |  |  |  |  |  |
|  | Physical activity plan | | | | | Yes | 1 | 1 | 1 | 1 | 1 | 1 |
|  |  |  |  |  |  | No | 2 | 2 | 2 | 2 | 2 | 2 |
|  |  | | | | |  |  |  |  |  |  |  |
|  | Healthy living plan | | | | | Yes | 1 | 1 | 1 | 1 | 1 | 1 |
|  |  |  |  |  |  | No | 2 | 2 | 2 | 2 | 2 | 2 |
|  |  | | | | |  |  |  |  |  |  |  |
|  | Plans for a gradual transition from work to retirement | | | | | Yes | 1 | 1 | 1 | 1 | 1 | 1 |
|  |  |  |  |  |  | No | 2 | 2 | 2 | 2 | 2 | 2 |
|  |  | | | | |  |  |  |  |  |  |  |
|  | Specific plans to maintain social activities after retirement | | | | | Yes | 1 | 1 | 1 | 1 | 1 | 1 |
|  |  |  |  |  |  | No | 2 | 2 | 2 | 2 | 2 | 2 |
| **Q.103** | If you have a retirement plan, at what age did you start seriously thinking about planning for your retirement? | | | | | |  |  |  |  |  |  |
|  |  | | | | In my 20s | | 1 | 1 | 1 | 1 | 1 | 1 |
|  |  | | | | In my 30s | | 2 | 2 | 2 | 2 | 2 | 2 |
|  |  | | | | In my 40s | | 3 | 3 | 3 | 3 | 3 | 3 |
|  |  | | | | In my 50s | | 4 | 4 | 4 | 4 | 4 | 4 |
|  |  | | Do/did not have a retirement plan | | | | 5 | 5 | 5 | 5 | 5 | 5 |
| **Q.104** | If help was available to advise on planning for a healthy, secure retirement, would you seek that advice? | | | | | |  |  |  |  |  |  |
|  |  | | | | | Yes | 1 | 1 | 1 | 1 | 1 | 1 |
|  | **GO TO Q.106🡺** | | | | | No | 2 | 2 | 2 | 2 | 2 | 2 |

|  | | | |  | | *Person 6* | | |  |  |  |  |  |  |
| --- | --- | --- | --- | --- | --- | --- | --- | --- | --- | --- | --- | --- | --- | --- |
| For each question, each household member is to **circle the number next to their response** | | | |  | | *Person 5* | | |  |  |  |  |  |  |
|  |  |  |  |  | | *Person 4* | | |  |  |  |  |  |  |
|  |  |  |  |  | | *Person 3* | | |  |  |  |  |  |  |
| **Jean Hailes Foundation (continued)** | | | |  | | *Person 2* | | |  |  |  |  |  |  |
|  |  |  |  |  | | *Person 1* | | |  |  |  |  |  |  |
| **Emotional wellbeing and relationships (continued)** | | | | | | | | |  |  |  |  |  |  |
| **Q.105** | [**IF YES**] From which of these sources? (**SELECT ALL THAT APPLY**) | | | | | | | |  |  |  |  |  |  |
|  |  | | | | | | | GP | 1 | 1 | 1 | 1 | 1 | 1 |
|  |  | | | | | | Pharmacist | | 2 | 2 | 2 | 2 | 2 | 2 |
|  |  | | | | | | Internet | | 3 | 3 | 3 | 3 | 3 | 3 |
|  |  | Attend an education session in my local area if available | | | | | | | 4 | 4 | 4 | 4 | 4 | 4 |
|  |  | | | | | | Magazines | | 5 | 5 | 5 | 5 | 5 | 5 |
|  |  | | | | | | Newspapers | | 6 | 6 | 6 | 6 | 6 | 6 |
|  |  | | Weight loss organisations (Weight Watchers etc) | | | | | | 7 | 7 | 7 | 7 | 7 | 7 |
|  |  | | | | | | Dietitians | | 8 | 8 | 8 | 8 | 8 | 8 |
|  |  | | | | Friends or family members | | | | 9 | 9 | 9 | 9 | 9 | 9 |
|  |  | | | | | | None of these | | 10 | 10 | 10 | 10 | 10 | 10 |
| **Q.106** | Are you caring for an aging parent with health problems? | | | | | | | |  |  |  |  |  |  |
|  |  | | | | | | | Yes | 1 | 1 | 1 | 1 | 1 | 1 |
|  |  | | | | | | | No | 2 | 2 | 2 | 2 | 2 | 2 |
| **Q.107** | Do you feel confident: | | | | | | | |  |  |  |  |  |  |
|  | That your parents will be adequately cared for in their old age through existing government services? | | | | | | | Yes | 1 | 1 | 1 | 1 | 1 | 1 |
|  |  |  |  |  |  |  |  | No | 2 | 2 | 2 | 2 | 2 | 2 |
|  |  | | | | | | |  |  |  |  |  |  |  |
|  | That you have a reasonable knowledge of support services available? | | | | | | | Yes | 1 | 1 | 1 | 1 | 1 | 1 |
|  |  |  |  |  |  |  |  | No | 2 | 2 | 2 | 2 | 2 | 2 |
|  |  | | | | | | |  |  |  |  |  |  |  |
|  | That your GP will be able and willing to provide support and information on care services when required? | | | | | | | Yes | 1 | 1 | 1 | 1 | 1 | 1 |
|  |  |  |  |  |  |  |  | No | 2 | 2 | 2 | 2 | 2 | 2 |
| **Q.108** | Would you like more information on available services for all Australians as we age. | | | | | | | |  |  |  |  |  |  |
|  |  | | | | | | | Yes | 1 | 1 | 1 | 1 | 1 | 1 |
|  | **GO TO Q.110🡺** | | | | | | | No | 2 | 2 | 2 | 2 | 2 | 2 |

|  | | | |  | | *Person 6* | | | |  |  |  |  |  |  |
| --- | --- | --- | --- | --- | --- | --- | --- | --- | --- | --- | --- | --- | --- | --- | --- |
| For each question, each household member is to **circle the number next to their response** | | | |  | | *Person 5* | | | |  |  |  |  |  |  |
|  |  |  |  |  | | *Person 4* | | | |  |  |  |  |  |  |
|  |  |  |  |  | | *Person 3* | | | |  |  |  |  |  |  |
| **Jean Hailes Foundation (continued)** | | | |  | | *Person 2* | | | |  |  |  |  |  |  |
|  |  |  |  |  | | *Person 1* | | | |  |  |  |  |  |  |
| **Emotional wellbeing and relationships (continued)** | | | | | | | | | |  |  |  |  |  |  |
| **Q.109** | [**IF YES**] From which of these sources? (**SELECT ALL THAT APPLY**) | | | | | | | | |  |  |  |  |  |  |
|  |  | | | | | | | GP | | 1 | 1 | 1 | 1 | 1 | 1 |
|  |  | | | | | | Pharmacist | | | 2 | 2 | 2 | 2 | 2 | 2 |
|  |  | | | | | | Internet | | | 3 | 3 | 3 | 3 | 3 | 3 |
|  |  | Attend an education session in my local area if available | | | | | | | | 4 | 4 | 4 | 4 | 4 | 4 |
|  |  | | | | | | Magazines | | | 5 | 5 | 5 | 5 | 5 | 5 |
|  |  | | | | | | Newspapers | | | 6 | 6 | 6 | 6 | 6 | 6 |
|  |  | | Weight loss organisations (Weight Watchers etc.) | | | | | | | 7 | 7 | 7 | 7 | 7 | 7 |
|  |  | | | | | | Dietitians | | | 8 | 8 | 8 | 8 | 8 | 8 |
|  |  | | | | Friends or family members | | | | | 9 | 9 | 9 | 9 | 9 | 9 |
|  |  | | | | | | None of these | | | 10 | 10 | 10 | 10 | 10 | 10 |
| **Q.110** | What are your fears with growing older? | | | | | | | | |  |  |  |  |  |  |
|  | Loneliness | | | | | | | | Yes | 1 | 1 | 1 | 1 | 1 | 1 |
|  |  |  |  |  |  |  |  |  | No | 2 | 2 | 2 | 2 | 2 | 2 |
|  |  | | | | | | | |  |  |  |  |  |  |  |
|  | Losing independence | | | | | | | | Yes | 1 | 1 | 1 | 1 | 1 | 1 |
|  |  |  |  |  |  |  |  |  | No | 2 | 2 | 2 | 2 | 2 | 2 |
|  |  | | | | | | | |  |  |  |  |  |  |  |
|  | Changing appearance | | | | | | | | Yes | 1 | 1 | 1 | 1 | 1 | 1 |
|  |  |  |  |  |  |  |  |  | No | 2 | 2 | 2 | 2 | 2 | 2 |
|  |  | | | | | | | |  |  |  |  |  |  |  |
|  | Losing loved ones | | | | | | | | Yes | 1 | 1 | 1 | 1 | 1 | 1 |
|  |  |  |  |  |  |  |  |  | No | 2 | 2 | 2 | 2 | 2 | 2 |
|  |  | | | | | | | |  |  |  |  |  |  |  |
|  | Physical health-related conditions, i.e. osteoporosis, glaucoma | | | | | | | | Yes | 1 | 1 | 1 | 1 | 1 | 1 |
|  |  |  |  |  |  |  |  |  | No | 2 | 2 | 2 | 2 | 2 | 2 |
|  |  | | | | | | | |  |  |  |  |  |  |  |
|  | Mental health-related conditions, i.e. dementia, depression | | | | | | | | Yes | 1 | 1 | 1 | 1 | 1 | 1 |
|  |  |  |  |  |  |  |  |  | No | 2 | 2 | 2 | 2 | 2 | 2 |
|  |  | | | | | | | |  |  |  |  |  |  |  |
|  | Finances/pension | | | | | | | | Yes | 1 | 1 | 1 | 1 | 1 | 1 |
|  |  |  |  |  |  |  |  |  | No | 2 | 2 | 2 | 2 | 2 | 2 |
|  |  | | | | | | | |  |  |  |  |  |  |  |
|  | Living in a retirement village or nursing home | | | | | | | | Yes | 1 | 1 | 1 | 1 | 1 | 1 |
|  |  |  |  |  |  |  |  |  | No | 2 | 2 | 2 | 2 | 2 | 2 |

|  | | | |  | | | | | *Person 6* |  |  |  |  |  |  |
| --- | --- | --- | --- | --- | --- | --- | --- | --- | --- | --- | --- | --- | --- | --- | --- |
| For each question, each household member is to **circle the number next to their response** | | | |  | | | | | *Person 5* |  |  |  |  |  |  |
|  |  |  |  |  | | | | | *Person 4* |  |  |  |  |  |  |
|  |  |  |  |  | | | | | *Person 3* |  |  |  |  |  |  |
| **Jean Hailes Foundation (continued)** | | | |  | | | | | *Person 2* |  |  |  |  |  |  |
|  |  |  |  |  | | | | | *Person 1* |  |  |  |  |  |  |
| **Emotional wellbeing and relationships (continued)** | | | | | | | | | |  |  |  |  |  |  |
| **Q.111** | What do you think are factors contributing to how long people live? (**SELECT ALL THAT APPLY**) | | | | | | | | |  |  |  |  |  |  |
|  |  | | | | | | | Environmental factors | | 1 | 1 | 1 | 1 | 1 | 1 |
|  |  | | | | Genetics (history of longevity in the family) | | | | | 2 | 2 | 2 | 2 | 2 | 2 |
|  |  | | | | | | Lifestyle (exercise and diet) | | | 3 | 3 | 3 | 3 | 3 | 3 |
|  |  | Having a disease prevention strategy including regular screening | | | | | | | | 4 | 4 | 4 | 4 | 4 | 4 |
|  |  | | | | | | Work environment/type of job | | | 5 | 5 | 5 | 5 | 5 | 5 |
|  |  | | | | | | Having satisfying hobbies | | | 6 | 6 | 6 | 6 | 6 | 6 |
|  |  | | | | | | Stable home life/relationships | | | 7 | 7 | 7 | 7 | 7 | 7 |
|  |  | | | | | | None of these/Don’t know | | | 8 | 8 | 8 | 8 | 8 | 8 |
| **Q.112** | As you get older do you think you spend— (**SELECT THE ANSWER THAT BEST DESCRIBES YOUR FEELINGS**) | | | | | | | | |  |  |  |  |  |  |
| More time concentrating on your own needs (e.g. your health and happiness) | | | | | | | | | | 1 | 1 | 1 | 1 | 1 | 1 |
| More time concentrating on other people’s needs (e.g. partner, children, parents) | | | | | | | | | | 2 | 2 | 2 | 2 | 2 | 2 |
|  |  | | About equal time on your needs and the needs of others | | | | | | | 3 | 3 | 3 | 3 | 3 | 3 |
|  |  | | | | | No family or close personal relationships | | | | 4 | 4 | 4 | 4 | 4 | 4 |
| ***That’s all the questions in this survey, thanks for your time.*** | | | | | | | | | | | | | | | |

| About this Survey— |  | |
| --- | --- | --- |
| Thank you for taking the time to complete the survey. We will be sending you another survey in mid–200- asking questions for different health care organisations. If you do not want to participate in further Pfizer Australia Health report surveys please tick the box below. |  | |
| I do not want to participate in further Pfizer Australia Health Report surveys: |  |  |
| We value the time and effort you have put into completing this survey. If you have any questions about completing the survey, about the reasons why we are asking these questions, about how the data is going to be used, or any other aspect of this project, please call our office on 1800 010 543. |  | |
